# *Pf*HRP2 detection using plasmonic optrodes: performance analysis

**DOI:** 10.1186/s12936-021-03863-3

**Published:** 2021-07-28

**Authors:** Médéric Loyez, Mathilde Wells, Stéphanie Hambÿe, François Hubinon, Bertrand Blankert, Ruddy Wattiez, Christophe Caucheteur

**Affiliations:** 1 Proteomics and Microbiology Department, University of Mons, Champ de Mars 6, 7000 Mons, Belgium; 2 Laboratory of Pharmaceutical Analysis, University of Mons, Avenue Maistriau 15, 7000 Mons, Belgium; 3 Electromagnetism and Telecommunications Department, University of Mons, Bld. Dolez 31, 7000 Mons, Belgium

**Keywords:** Optical fibers, Malaria diagnosis, *Plasmodium falciparum*, HRP2, LDH, SPR, Biosensing

## Abstract

**Background:**

Early malaria diagnosis and its profiling require the development of new sensing platforms enabling rapid and early analysis of parasites in blood or saliva, aside the widespread rapid diagnostic tests (RDTs).

**Methods:**

This study shows the performance of a cost-effective optical fiber-based solution to target the presence of *Plasmodium falciparum* histidine-rich protein 2 (*Pf*HRP2). Unclad multimode optical fiber probes are coated with a thin gold film to excite Surface Plasmon Resonance (SPR) yielding high sensitivity to bio-interactions between targets and bioreceptors grafted on the metal surface.

**Results:**

Their performances are presented in laboratory conditions using PBS spiked with growing concentrations of purified target proteins and within in vitro cultures. Two probe configurations are studied through label-free detection and amplification using secondary antibodies to show the possibility to lower the intrisic limit of detection.

**Conclusions:**

As malaria hits millions of people worldwide, the improvement and multiplexing of this optical fiber technique can be of great interest, especially for a future purpose of using multiple receptors on the fiber surface or several coated-nanoparticles as amplifiers.

**Supplementary Information:**

The online version contains supplementary material available at 10.1186/s12936-021-03863-3.

## Background

Malaria hits millions of people every year (228 million malaria cases worldwide in 2018) resulting in considerable number of deaths (405.000 in 2018), according to the World Health Organization (WHO) [1]. This parasitic disease is caused by several species of *Plasmodium* protozoa and can be transmitted by the bite of the female of more than thirty species of anopheline mosquitoes. The most common symptoms of *Plasmodium* spp infections are high fevers followed by chills and rigors by repeated cycles [2]. Severe cases can develop and lead to extreme anaemia, pulmonary, hepatic and cerebral forms. Some species can also stay dormant in the liver upon inoculation and cause the recurrence of the disease. Malaria remains endemic in specific geographic areas, but thanks to the recent technological advances and large efforts invested in research and aid to developing countries, the hope for its eradication by 2030 becomes a real perspective [3].

Towards this malaria eradication, different and complementary paths need to be explored. For instance, the interruption of local transmissions is urgent because it is correlated with the rapid management of patients and the quality of care and treatment. This is often lacking in poor countries, and limits the decline in new infections. Diagnostics are usually performed using rapid diagnostic tests (RDTs) which are numerous and varied. Most of them rely on the detection of specific antigens released in blood or saliva by parasites. Some of them mainly focus on one species (such as *Plasmodium falciparum* which is responsible of more than 99% of cases in some regions), while others are multiplexed for the detection of other *Plasmodium* species (*Plasmodium vivax, Plasmodium malariae* and *Plasmodium ovale).* RDTs are mainly based on lateral flow immunochromatographic assays to detect free-circulating biomarkers[4]. They target histidine-rich protein 2 (*Pf*HRP2) proteins using monoclonal antibodies, as HRP2 is present in abundance during the asexual cycle and in early gametocyte stages of *P. falciparum* parasites [5, 6]. Other biosensors also target the lactate dehydrogenase (*p*LDH). While *Pf*HRP2 may remain detectable up to several days or even weeks after clearance of the disease, *p*LDH is a survival metabolic enzyme produced by the five species of parasites and does not persist after the infection has cleared. A combined detection of these two proteins could, therefore, be of interest to determine if a treatment is needed or if clearance already occurred [7, 8].

Among all the biosensors available to detect these proteins, immunosensors and especially paper-based dipsticks are probably the most spread tools, with several analytical benefits and cost efficiency [9]. They are based on a simple concept with low production costs, immediate readout, and remain the main solution for mass diagnostics on the field. However, they are often used late and their LOD (best-in-class RDTs are around 0.8 ng/mL for *Pf*HRP2 or ~ 200 parasites/µL) does not always allow diagnostic at low parasitaemia levels, leading to false negative issues in very specific cases (early infections or asymptomatic cases) [10]. The effective threshold for the detection of biomarkers using RDTs is not straightforward to correlate with blood tests if not directly calibrated by the manufacturer, as the link between the HRP2 blood level and parasitaemia of infected patients is weak [11].

It is also important to mention that many studies call on microscopy for the quantification of malaria parasites in blood. Microscopy is a direct method for the detection of parasites, but it is user-dependent, poorly reproducible, and it often reveals false-positive results. There is, therefore, room for improvement in applied biosensing, as high-resolution systems are often limited to laboratory use and not compatible with low-cost portable platforms, which is of prime importance in remote places with little access to new technologies. This being said, RDTs are commercialized at large-scale and aim at diagnosing malaria directly on site. On the other hand, a growing interest on optical fiber (OF) sensors has arisen, and multiple research platforms have proven their interest to finely sense biomarkers at ultralow levels. Among all OF architectures available, unclad fibers remain the most practical platform towards a simple implementation for cost-effective and online monitoring. This configuration is, therefore, studied in this work.

Last but not least, a fair regulation between commercial diagnostic tests and availability of treatment and care is also needed. In fact, the race for the eradication of malaria faces with an increase in drug resistance (especially artemisinin resistance) due to unregulated treatment. It is, therefore, essential that patients are efficiently tested positively before taking anti-malarial drugs to avoid this phenomenon and allow current therapies to remain effective [12].

In this article, a lab-on-fiber platform for malaria diagnosis is studied. OF probes (optrodes) are unclad silica multimode fibers (400 µm core diametre). They present one centimetre-long gold-coated tips with roughly 50 nm of gold deposited on their outer surface to excite surface plasmons (SPR) in the visible wavelength range. They are connected to a portable white light source and a spectrometer for read-out. This configuration allows the acquisition of SPR spectra, that are analysed for further biological detection while bioreceptors are grafted on their surface. This method corresponds to the transposition of the commercially-available Kretschmann prism configuration to unclad OFs [13]. Specific structures can be implemented to excite SPR with OFs such as the use of Tilted Fiber Bragg Gratings (TFBGs) [14–17], Long Period Fiber Gratings (LPFGs) [18, 19], U-bent fibers [20, 21], and many other lab on fiber (LOF) architectures [22, 23]. These probes have already proven their high potential for biomarkers detection in very different media, especially inside human tissues [24, 25] and body fluids [26, 27] among others [28, 29]. In this evolution to develop and evaluate new molecular detection techniques for malaria, the behaviour of our sensors is studied in different conditions to test their response and sensitivity against *pf*HRP2 biomarkers.

This original experimental study on *Plasmodium falciparum* cultures could open the door towards the integration of these OF probes into smaller biochips or bio-sticks connected to smartphone or specifically designed microfluidic packages. This could be of great interest for an innovative, portable and cost-effective diagnosis method [30–32].

## Methods

### Materials

Phosphate Buffer Saline (PBS) came from Thermo Fisher Scientific (Waltham, MA, USA). 6-mercapto-1-hexanol was purchased from Sigma-Aldrich (Merck, Darmstadt, Germany). The HRP2 proteins were purchased from Thermo Fischer Scientific. FT400UMT TECS Hard Clad, 0.39 NA, Step-index OF came from Thorlabs. Vytran CAC-400 compact fiber cleaver, the 400 µm Y-bundle FC/PC and BFT1 connectors came from Thorlabs. Halogen white light source and HR4000 spectrometers came from OceanOptics. (Trimethylsilyls)-3-propanethiol came from VWR. Amine coupling kit (NHS/WSC, activation buffer and blocking BSA 1%) came from Dojindo Molecular Technologies, Inc. (GERBU Biotechnik GmbH, Germany). Laboratory-adapted *P. falciparum* strain 3D7 was purchased from BEI Resources (VA, USA). The RPMI 1640 powder supplemented with Hepes and L-Glutamine, as well as the AlbuMax™ II and Gentamicin (50 mg/mL) were purchased from Thermo Fisher Scientific (Gibco®, Waltham, MA, USA). Hypoxanthine and sodium bicarbonate were purchased from Sigma-Aldrich (Merck, Darmstadt, Germany). Concentrated red blood cells were provided by the Red Cross Organization (Namur, Belgium). ELISA kit for the detection and quantification of HRP2 (Ultra-Sensitive Quantimal Celisa KM8/KM8BP) came from Cellabs (Brookvale, Australia).

### Optical fibers preparation

**Fig. 1:**
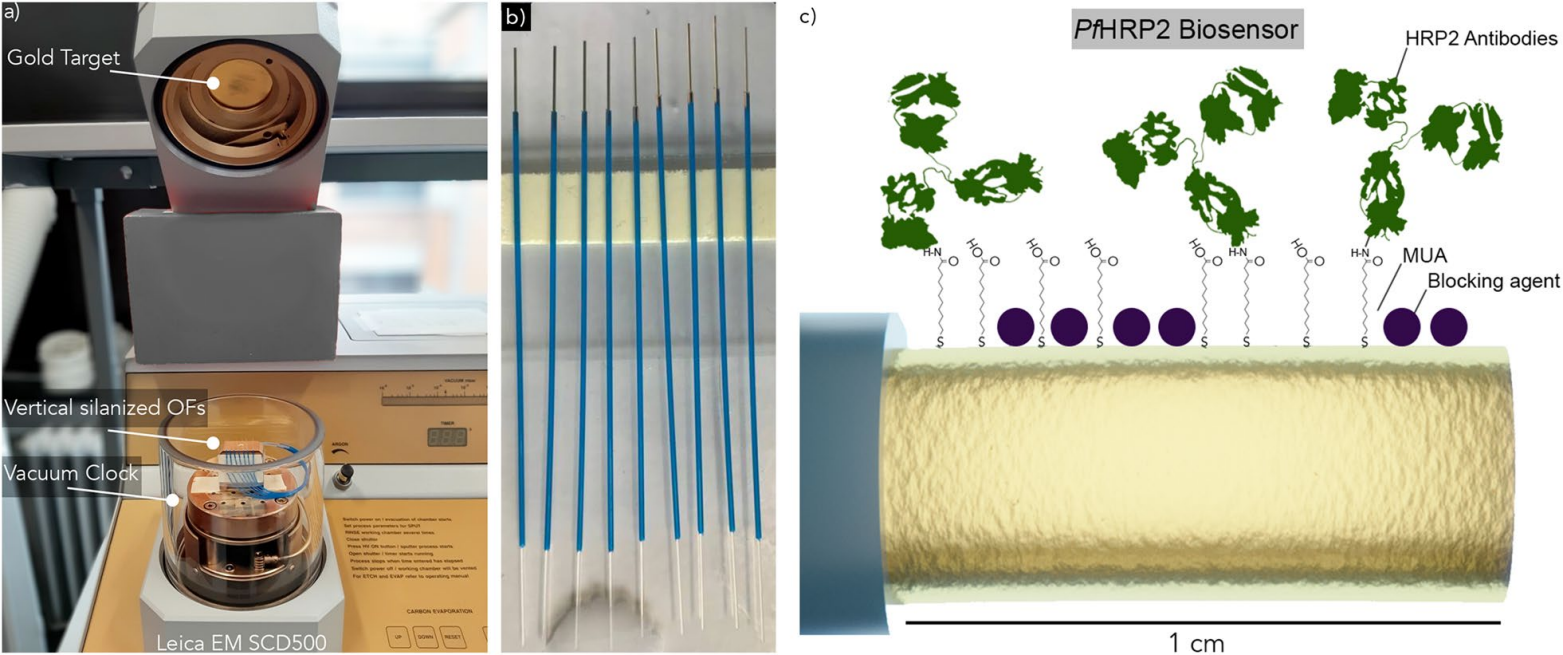
**a** Picture of the gold sputter-coater. OFs are placed vertically inside the vacuum chamber. The quartz microbalance is at the level of the lower stage in the vacuum clock. **b** Picture of the prepared 400 µm diametre OFs, ready for the functionalization process. **c** Scheme of the biochemical functionalization process with covalent coupling of HRP2 antibodies and implementation of the blocking agent

The OF-SPR sensors tips were manufactured using FT400 Step-Index multimode fiber with a core diameter of 400 µm (Thorlabs). Pieces of 15 cm OF were cut, and a 4 cm end was stripped using a clamp. The stripped part of the OF was then immersed in acetone to remove the TECS hard polymer cladding, and cleaned manually by a cloth soaked in acetone until total removal of cladding remains. OFs were then cleaved at the right angle using a Vytran compact fiber cleaver (Thorlabs), to obtain 1 cm unclad sensing areas, and cut on the other side to connect the fiber. Final probe lengths of about 5 cm were used to avoid fiber bending during measurements. OF tips were then cleaned into piranha solution (H_2_SO_4_ and H_2_O_2_ 3:1) and silanized in methanol using 1% solution of (trimethylsilyls)-3-propanethiol during 15 min, at room temperature (RT). OFs were then dried overnight at RT and mounted on a holder for a single 50 nm gold sputter deposition, which was performed vertically (Fig. 1a, b). The fibers were then directly stored in cleaned Petri dishes on a holder to avoid any dusts contaminations of the fiber tip.

### Bio-functionalization

The immobilization of antibodies on the OFs as bioreceptors against *Pf*HRP2 was performed through covalent bonding (Fig. 1c)*.* OFs were immersed during 16 h in closed chambers of mercaptoundecanoic acid 2 mM in absolute ethanol at room temperature (RT). They were then washed in absolute ethanol and placed into 125 µL vials of 50 mM WSC and 50 mM NHS in activation buffer during 10 min at RT. They were then washed with PBS and immersed during 1h30 in anti-*Pf*HRP2 antibodies. The blocking solution was then applied after another rinsing with PBS during 30 min at RT. Finally, the fibers were washed again in PBS and dried for biodetection experiments.

### Interrogation setup and data analyses

**Fig. 2:**
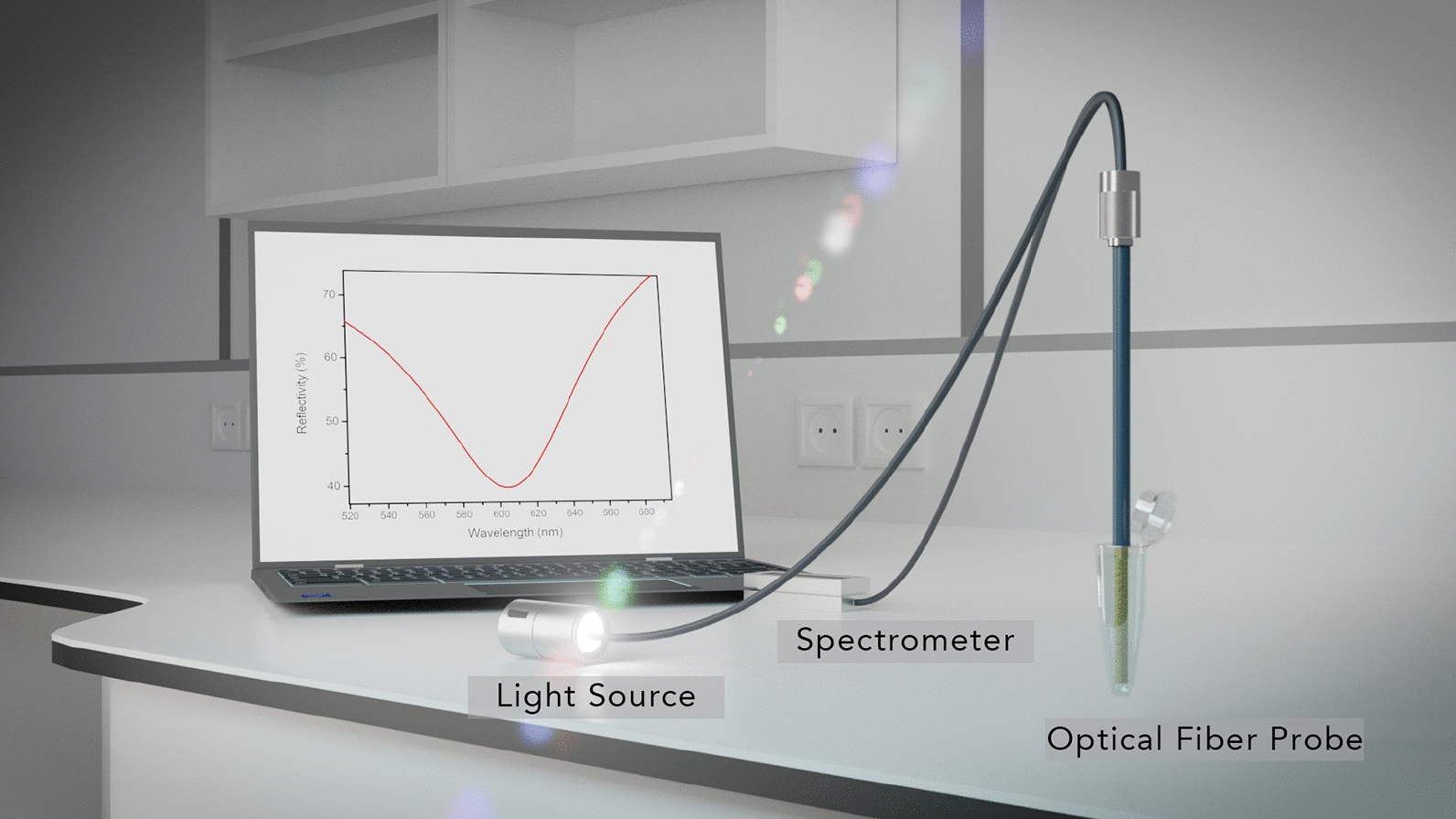
Artistic view of the experimental setup with its plasmonic response

OFs are connected through a bifurcator (Y-bundle FC/PC) to a white light source (halogen) and a spectrometer (Ocean Optics HR4000, with 500–900 nm range and resolution of 0.19 nm). The spectrum released with this OF-SPR technique results in a gaussian curve (Fig. 2) where the dip quality (full width at half maximum: FWHM and intensity: depth) is influenced by the mode propagation angle and the number of reflections occurring inside the OF [33]. Data were analysed through an in-house developed MATLAB program.

### *Plasmodium falciparum* cultures and sample preparation

Laboratory-adapted strains 3D7 were maintained in continuous culture following the protocol proposed by Trager and Jensen with some modifications. Parasites were cultured in complete medium supplemented at 5% haematocrit (A Rh^+^). Briefly, complete medium was prepared from RPMI 1640 powder (25 mM Hepes, 2 mM L-Glutamine) supplemented with 25 mM NaHCO_3_, 0.5% AlbuMax™ II, 2 mM Hypoxanthine and 0.002% Gentamicin. Cultures were incubated at 37 °C under humid atmosphere in a gas mixture containing 5% CO_2_. Cultures were diluted with fresh red blood cells every other day [34].

For the purpose of sample preparation, cultures were synchronized 48 h prior to sample collecting to have a majority of ring-stage forms. The sorbitol method was used to achieve synchronicity. Both whole culture and culture supernatant were used for analysis. Briefly, whole cultures were incubated with a 5% sorbitol solution at 37 °C for 15 min. The lot then underwent successive centrifugation cycles and finally put back in continuous culture as described previously. Two days post synchronization, cultures were left to sediment for 90 min in the incubator. After this period, the supernatant was taken up without disturbing the red blood cell layer. The supernatant was centrifuged at 3260 g for 5 min to remove any red blood cell residues. Whole culture samples were taken up and did not undergo any specific treatment before being analysed. Parasitemia and ring-stage percentage were evaluated by optical microscopy (Additional file 1: 1). Blood smears were prepared for each culture and stained (Giemsa 10%, × 1000). Parasitaemia was calculated as follows: [(number of infected red blood cells)/(total number of counted red blood cells)] × 100. Percentage of ring-stage was determined as the [(number of ring-stage infected red blood cells)/(total number of infected red blood cells)] × 100 [35].

### Validation using RDTs and ELISA

The presence of HRP2 proteins in whole culture and supernatant was first qualitatively verified using rapid diagnostic tests before the OF experimentation (Malaria Ag Pf/Pan from Bioline). The HRP2 proteins spiked in PBS were also tested to verify their detection. These RDTs report a sensitivity of 99.7% and a specificity of 99.5%. 5 µL- sample was placed on the round well and diluted with four drops of the included assay diluent. The result was read after 15 min, when the migration of the sample on the colored bands was completed. The color of the control line attests the good migration while the apparition of a second band attests the presence of HRP2 proteins. HRP2 quantification in cultures analysed by the OFs was performed afterwards through an ELISA. Two distinct calibration curves were established using the HRP2-purified protein spiked into the blank culture medium or the culture medium supplemented at 5% haematocrit at different concentrations. The samples were diluted 1:1000 using culture medium or control blood and incubated on the microtitre plate in triplicates following the supplier recommendations.

## Results and discussion

### Validation of the biosensor functionalization process

**Fig. 3:**
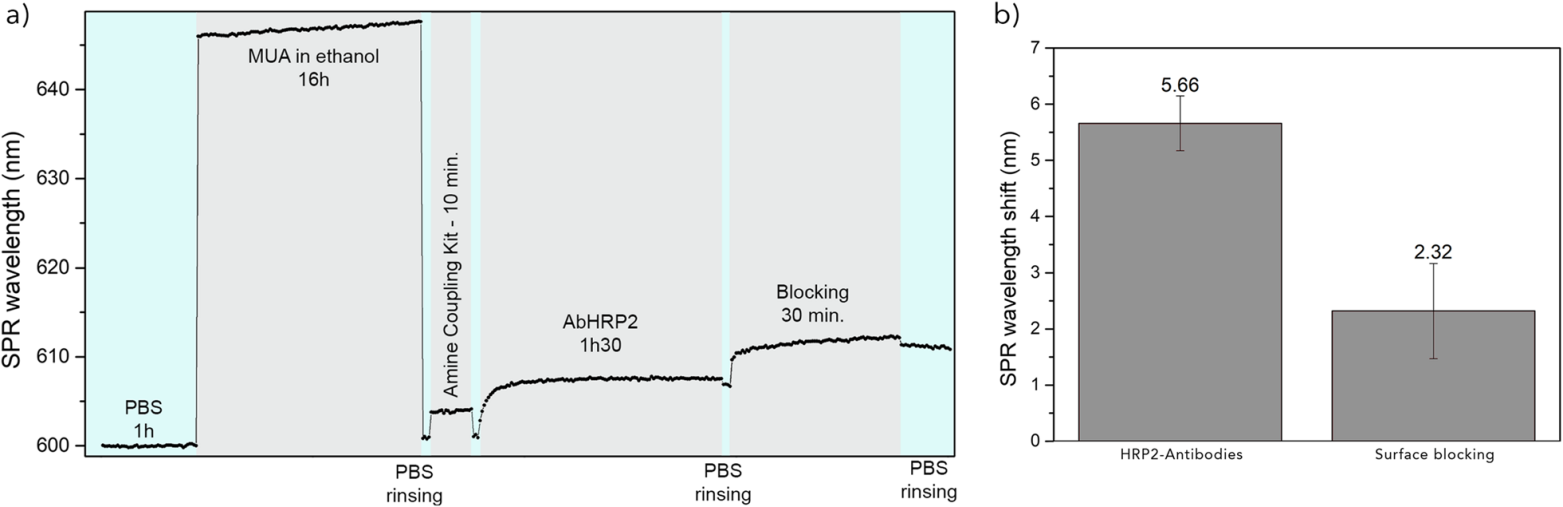
**a** Sensorgram for the biofunctionalization in MUA, NHS/EDC, HRP2 antibodies, and casein blocking (shifts of the SPR-dip wavelength). **b** Mean ± sd of the SPR wavelength shift obtained inside antibodies and blocking, after 90 and 30 min, respectively (n = 6)

Prior to biodetection experiments, the functionalization of the OFs and the related immobilization of the *pf*HRP2 antibodies was monitored in real time. First, the probe stability was verified into PBS during 1 h. The fibers were then immersed overnight into MUA 2 mM in ethanol to form a self-assembled monolayer (SAM) on the gold film. MUA molecules first bound to the surface and then self-organized, leading to a progressive shift of the SPR signal. The fibers were then rinsed and immersed into the solution of antibodies at a concentration of 20 µg/mL during 1h30. Finally, the blocking was also monitored (Fig. 3). This functionalization process shows the efficiency of the receptors binding to the fiber surface. This validation was performed for every lots of biosensors further tested. All these steps were performed in closed vials to avoid evaporation or perturbation of the medium during measurement, such as vibrations of the fiber probe.

### Optimization of the blocking process to work in complex matrices

**Fig. 4:**
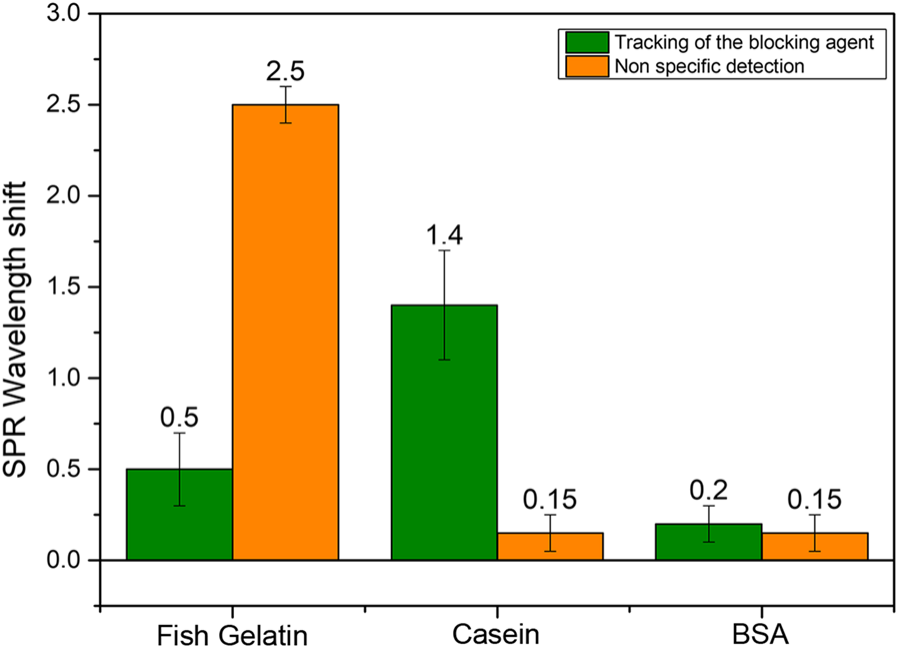
Blocking process monitoring and efficiency evaluated by the SPR wavelength shift during blocking and immersion of the optical fibers into culture medium (mean ± sd, n = 4 different fibers tested for each condition)

Live monitoring biosensors such as SPR-based platforms have to deal with non specific bindings occurring into complex media. Such unwanted interactions lead to false positive detections or block the interaction sites on the surface. To avoid these side effects, the blocking reaction was optimized by testing three well known methods, often used for immunoblotting or ELISA. The efficiency of three blocking agents was tested on top of gold-plated OFs using casein 0.1%, fish gelatin 0.1% and BSA 1% (m/V), adapted from different protocols reported in the literature [36–38]. The fibers were functionalized using MUA, the amine-coupling kit, and blocked using these procedures. The sensors were then immersed in PBS to check the stability of the probes and verify the strong anchoring of the surface biolayer. Afterwards they were immersed in culture medium, where an efficient blocking should avoid any perturbations of the signal. The fibers were then rinsed and immersed back into PBS. The first measurements show that the fish gelatin is not sufficient enough to block the surface while BSA and casein present better performances. The casein blocking agent also leads to a better stability in both PBS and the culture medium (Fig. 4). That method was, therefore, selected for further biosensing analyses. The latter is also easier to track during the immobilization process than the BSA, probably because its anchoring on the fiber surface provokes a higher surface refractive index shift, due to molecular sizes and weight differences or by its organization on the probe (homogeneity, surface shape). The organization of the blocking layers onto the surface may play a crucial role for the process monitoring and for the protection against non-specific adsorption.

### Detection of spiked PfHRP2 using optial fiber biosensors

**Fig. 5:**
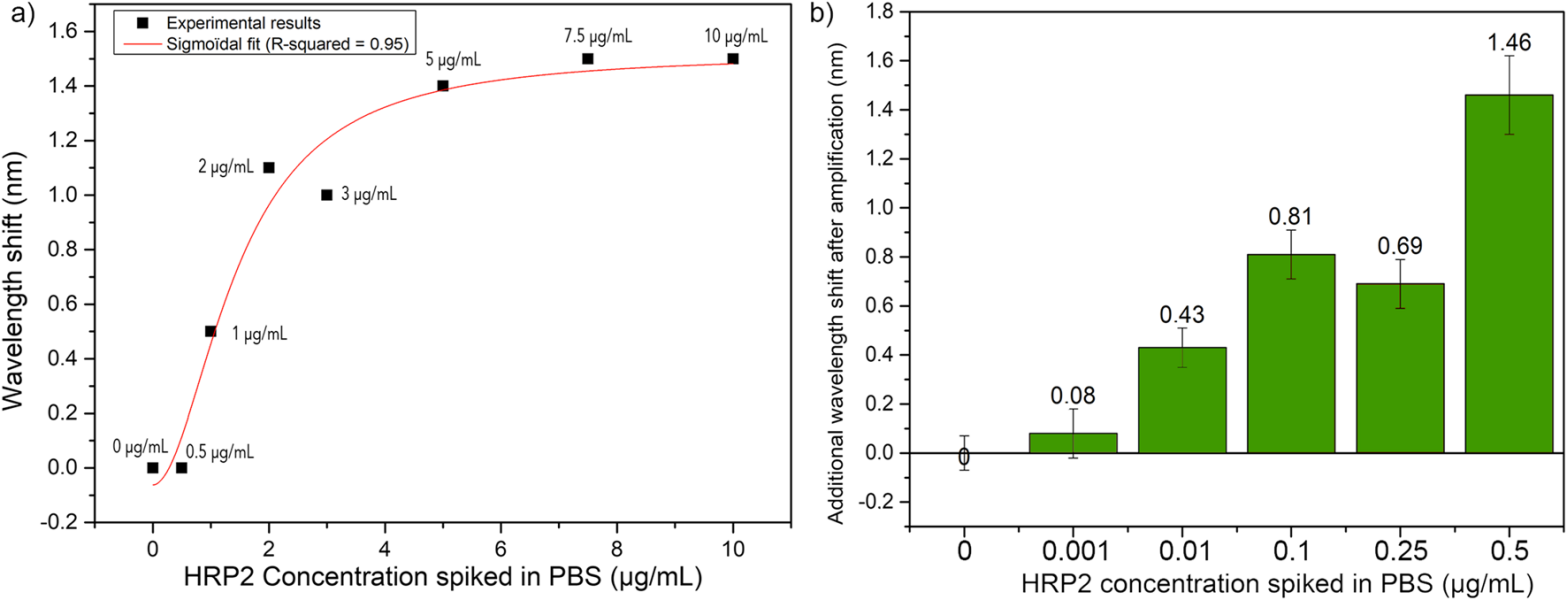
**a** Label-free detection of HRP2 spiked in PBS at different concentrations (each experimental point represents the data obtained by a different optical fiber biosensor). **b** Shift observed after the addition of antibodies as amplification tags (mean ± sd, n = 3 different fibers for each concentration)

OFs were immersed into PBS spiked with growing concentrations of *Pf*HRP2. First, the fibers were placed into blank PBS and then into PBS + HRP2. The shift was monitored in real time and the final detection was considered 5 min after the immersion of the probe. These shifts are reported in Fig. 5a. The detection starts at around 1 µg/mL in a label-free configuration. However, the needed LOD is often below that level, so that a sandwich bioassay should be foreseen. For instance, the use of coupled nanoparticles or secondary binders are often used to enhance the signal shift due to their higher effects on the surface refractive index. In this study, secondary antibodies were implemented to bind to the anchored HRP2 proteins in a sandwich-like assay. Using these amplifiers, the detection threshold was improved as shown on Fig. 5b where the signal shift after amplification begins at lower concentrations, which were not detected with the label-free configuration. Here, a significant plasmonic shift starts at around 100 ng/mL.

### Detection of HRP2 in culture supernatant and whole culture

**Fig. 6:**
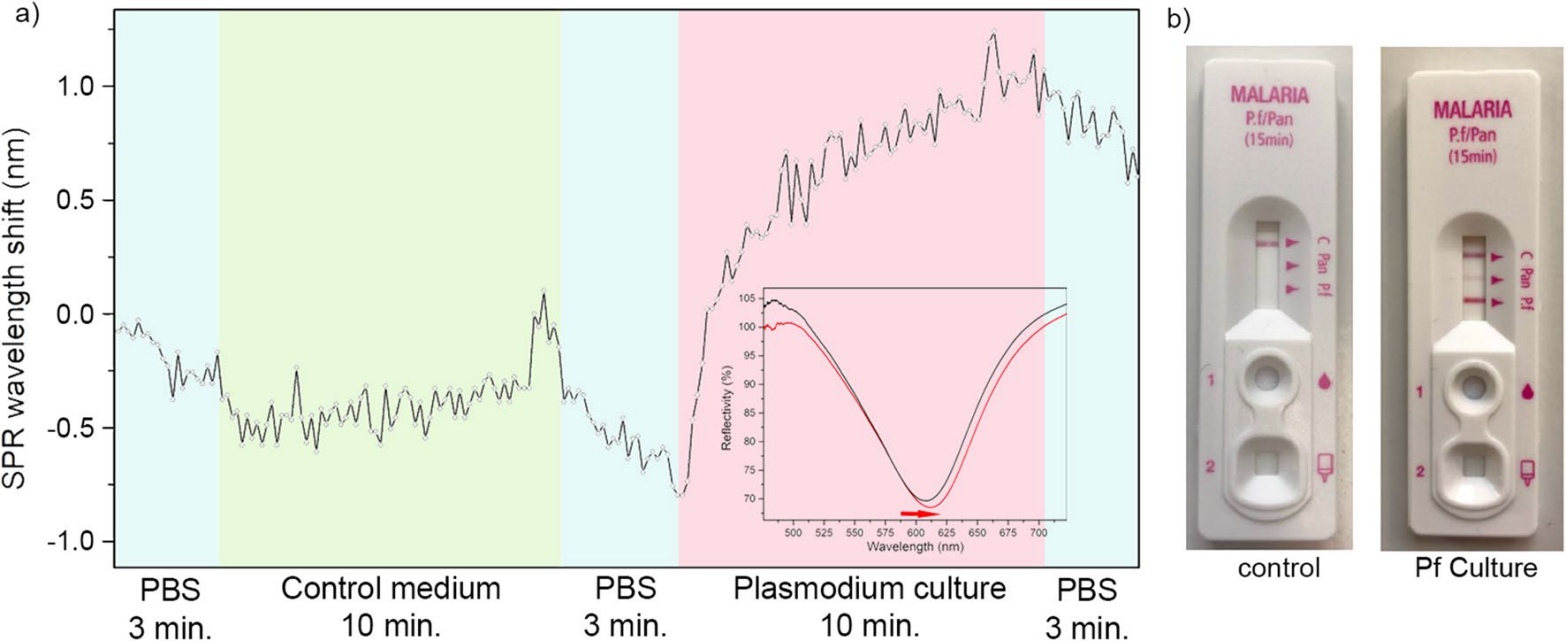
**a** Sensorgram showing the live SPR response in both control medium and *Plasmodium* culture containing HRP2 proteins, **b** as verified by a commercial strip test

The detection of HRP2 proteins was finally tested in vitro using cultures of *P. falciparum*. First, the optical fiber probes were immersed in PBS and then in cultures at 5% Ht without parasites (control) to verify their specificity but also to determine its starting SPR wavelength, as it is driven by the effective refractive index of the solution. There was no significant red shift observed in the control medium within 10 min. Its own refractive index is indeed close to the one of our PBS buffer which is equal to 1.3367 (given at 589 nm). The plasmonic probes were then immersed back in PBS to clean their surface and were immersed in *Plasmodium* culture for 10 min. A progressive red shift in the upper wavelengths of the SPR signal was observed, as presented in Fig. 6a. The fibers were then rinsed and disinfected into ethanol.

The detection of HRP2 inside these tested cultures was also attested by a strip-test showing the presence of the target by the apparition of a red band (Fig. 6b). An ELISA assay was also performed afterwards to quantify the amount of proteins in these cultures (Additional file 1: 2). It appears that the whole cultures tested show a concentration of HRP2 often ranging around 1 µg/mL while its supernatant following centrifugation has a much lower HRP2 concentration. It also seems impossible to precisely link the parasitaemia to the HRP2 expression, as it is often pointed out in the literature. Depending on the culture-time and synchronization, both parasitaemia and concentrations of HRP2 can be highly impacted.

### Discussion of the performances and tunability of the lab-on-fiber technology

Lab-on-fiber technology has shown major advances over the last decade [39, 40]. The unclad fiber approach presented in this article also remains a practical method to make use of plasmonics inside low-volume samples and to quicken the acquisition time compared to routine assays such as the ELISA [41]. It leads to highly tunable OFs that are interesting for many applications, especially to monitor live molecular events. In this paper, biofunctionalized optrodes were studied and optimized for the detection of HRP2 proteins in the framework of malaria detection. The possibility to use amplifiers to lower the LOD was shown, and the fibers were tested in complex media such as in vitro cultures of *P. falciparum*. The performances reached in this paper still leave room for improvements, especially to achieve more user-friendly interfaces. However, the many assets of OFs and their ease of implementation would enable substantial alternatives to the traditional equipment currently used at a research level, for instance, their combination with smartphones and portable devices, the elaboration of online bioprofiling databases [30].

The use of other molecular amplifiers to improve the LOD and reach the performances of best-in-class RDTs can also be investigated and optimized, such as the coupling of gold nanoparticles or nano-shell particles, often put in evidence in this field of research [42, 43]. The use of more specific targets such as *p*LDH or other bioreceptors, such as DNA aptamers or molecularly imprinted polymers (MIPs) could also be investigated to improve biosensing responses [44].

## Conclusion

In this work, the detection of HRP2 proteins with unclad OF probes through plasmonic assays was performed. By anchoring antibodies on the fiber surface, it was possible to monitor the presence of the target proteins by a label-free configuration (down to 1 µg/mL) while its enhancement by the affinity of secondary binders can improve the sensor sensitivity by a factor of 10. The detection was also performed on *Plasmodium* cultures, where HRP2 levels were tested by commercially available strip tests and a quantitative ELISA. As malaria affects many people around the world, improvement and multiplexing of this technique can be of great interest, especially because of its high tunability, both in terms of surface biochemistry and in terms of live spectral acquisition. The use of multiple receptors on the fiber surface or several coated-nanoparticles as amplifiers could still improve its LOD and lead to a practical tool, e.g. to discriminate the parasite causing malaria and early diagnosis purposes.

## Supplementary Information

**Additional file 1: 1.** Optical microscopy of Plasmodium falciparum cultures. **2.** Detection of PfHRP2 through ELISA.

## Data Availability

The dataset(s) supporting the conclusions of this article is(are) included within the article (and its additional file(s)).
