# Supplementary material for: PfHRP2 detection using plasmonic optrodes: performance analysis
Source: Malar J. 2021 Jul 28;20:332. doi: 10.1186/s12936-021-03863-3 (PMC8320217; doi:10.1186/s12936-021-03863-3)
Supplement: Supplementary file 1 — Additional file 1: 1. Optical microscopy of Plasmodium falciparum cultures. 2. Detection of PfHRP2 through ELISA. [file 12936_2021_3863_MOESM1_ESM.docx]

***Pf*HRP2 detection using plasmonic optrodes:**

**performances analysis**

Médéric Loyez^1^, Mathilde Wells^2^, Stéphanie Hambÿe^2^, François Hubinon^3^,
Bertrand Blankert^2^, Ruddy Wattiez^1^, Christophe Caucheteur^3^

^1^ Proteomics and Microbiology Department, University of Mons, Champ de Mars 6, 7000 Mons, Belgium.

^2^ Laboratory of Pharmaceutical Analysis, University of Mons, Avenue Mestriaux 15, 7000 Mons, Belgium.

^3^ Electromagnetism and Telecommunications Department, University of Mons, Bld. Dolez 31, 7000 Mons, Belgium.

1. **Optical microscopy of *Plasmodium falciparum* cultures**


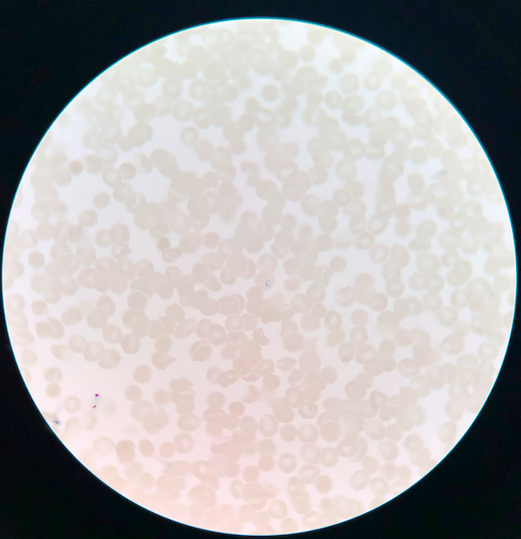

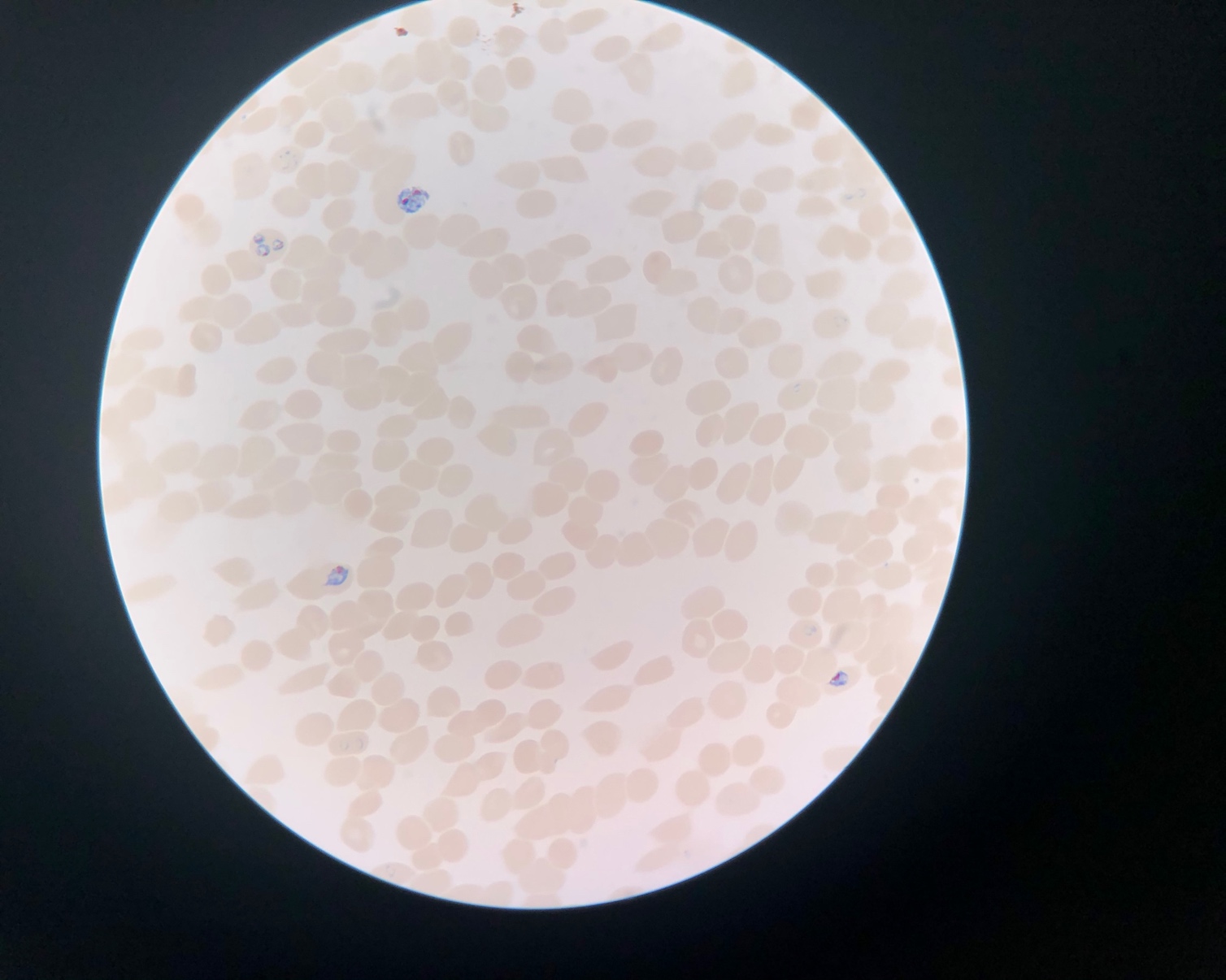


a)

c)


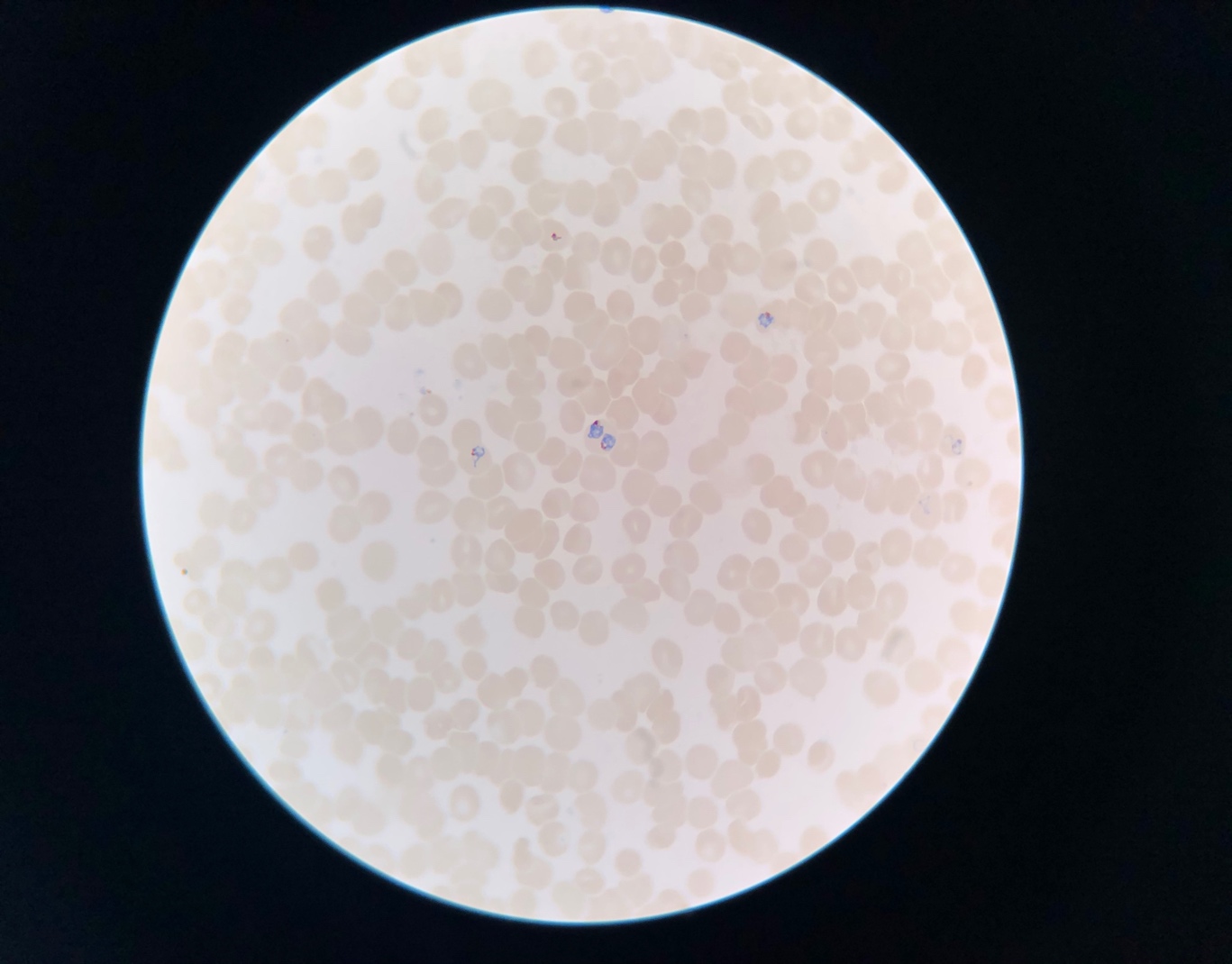


b)

***Figure S1.*** *Microscopic slide captions of thin blood smears from continuous cultures of Plasmodium falciparum (3D7 strain), Giemsa stain (1:100), Magnification x1000. a) Ring-stage; b) Trophozoite stage; c) Schizont stage.*

1. **Detection of PfHRP2 through ELISA**

| Culture sampling  (date) | % Parasitemia (microscopy) | HRP2 concentration  ng/mL (ELISA) |
| --- | --- | --- |
| 12/08 | 0.44 | Supernatant: 31.26 |
| 20/08 | 1.61 | Supernatant: 0.66 whole culture: 894.27 |
| 31/08 | 0.23 | Supernatant: 204.06 whole culture: 221.24 |
| 09/10 | 1.25 | Supernatant: 75.412 whole culture: 1292.206 |
| 21/10 | 0.90 | Supernatant: 28.73 whole culture: 1065.69 |
| 28/10 | 0.84 | whole culture: 1265.34 |
